# Autonomous disproofs of the Erdős unit distance conjecture with GPT-5.5 Pro

**DOI:** 10.1073/pnas.2621541123

**Published:** 2026-10-26

**Authors:** Yichen Huang

**Affiliations:** ^a^Santa Clarita, CA 91350

## Abstract

OpenAI’s disproof of the Erdős unit distance conjecture marks a milestone in AI for mathematical research. However, the result relied on an internal model with undisclosed test-time compute, hindering independent reproduction and analysis by the community. Here we present a simple autonomous agent built on the publicly available GPT-5.5 Pro. The agent uses a problem-agnostic three-stage prompting pipeline: brainstorm, execute, and review. We ran eight independent trials with web search disabled. In each trial, the agent produced a correct or essentially correct disproof of the conjecture. The resulting proofs are diverse and do not merely reproduce earlier AI-generated proofs. Since GPT-5.5 Pro was released nearly a month before OpenAI published its disproof, our evaluation is free from data contamination. The system operates efficiently, using an average of 106.7 k reasoning tokens per trial. We release our code and all outputs, providing a publicly accessible AI agent to resolve this conjecture autonomously.

In May 2026, OpenAI announced (1) that an internal AI model autonomously disproved the Erdős unit distance conjecture.^*^ This achievement marks a milestone in AI for mathematical research, demonstrating that AI systems are now capable of resolving significant, decades-old open conjectures.

## The Erdős Unit Distance Conjecture.

The unit distance problem was introduced by Erdős in 1946 (3). It asks for the maximum number of unit-distance pairs, u(n), that can occur within a set of *n* points in the Euclidean plane. Erdős established a lower bound of $n^{1+\Omega (1/\log\;\log\;n)}$ using a scaled square grid, and conjectured the upper bound $n^{1+o(1)}$. For decades, the mathematical community largely believed Erdős’s conjecture to be true. Erdős also proved an upper bound of $O(n^{3/2})$ by observing that the unit distance graph cannot contain the complete bipartite graph $K_{2,3}$ as a subgraph, since any two distinct unit circles intersect in at most two points (3). The best known upper bound, $O(n^{4/3})$, was proved by Spencer et al. in 1984 (4), with a subsequent shorter proof by Székely (5).

The OpenAI proof (6) refuted the conjecture by showing that there exists an absolute constant δ>0 such that $u\left(n\right)\geq n^{1+\delta }$ for infinitely many positive integers *n*. Rather than using geometric or combinatorial methods directly in two dimensions, the proof relies on algebraic number theory. It uses a sequence of number fields whose degrees tend to infinity to construct lattices in higher-dimensional Euclidean spaces. Carefully chosen lattice points are then mapped to the plane, yielding point sets with sufficiently many unit-distance pairs. Subsequent human work simplified the AI-generated proof (7) and established the explicit lower bound $u\left(n\right)\geq n^{1.014}$ for infinitely many *n* (2).

## Subsequent Developments.

Following OpenAI’s breakthrough, Alpöge (8) announced that an agent based on Anthropic’s Claude Mythos autonomously disproved the conjecture, yielding a different proof. Although this proof explicitly established the weaker lower bound $u\left(n\right)\geq n^{1+\frac{\log\;\log\;\log\;n}{192\;\log\;\log\;n}}$ for infinitely many *n*, Alpöge clarified in a footnote (9) that a minor modification to the proof yields the stronger bound $u\left(n\right)\geq n^{1+\delta }$. However, because neither OpenAI’s internal model nor the Claude Mythos agent is publicly available,^†^ the broader research community lacks the access needed to independently reproduce these autonomous runs. Although OpenAI published success rates (pass@1 accuracy), the absolute amount of test-time compute remains undisclosed (Fig. 1). For the Claude Mythos agent, neither pass@1 accuracy nor test-time compute has been reported.

**Fig. 1.**
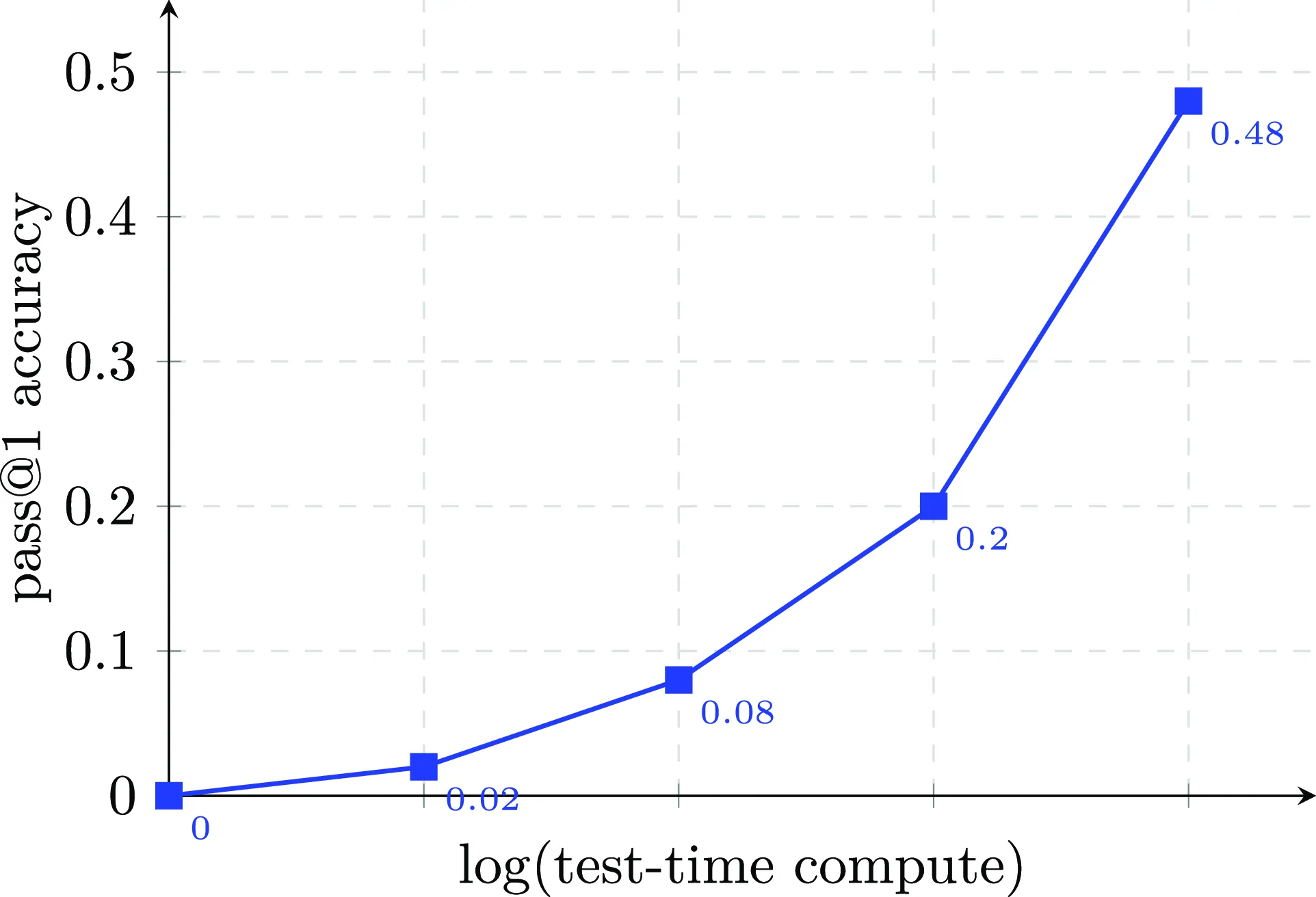
Test-time scaling curve for OpenAI’s internal model, reproduced from ref. 1. The horizontal axis represents the logarithm of test-time compute (with the exact scale undisclosed). The vertical axis represents pass@1 accuracy. The pass@1 accuracy reaches 0.48 at the maximum test-time compute reported by OpenAI.

Attempts to disprove the conjecture using publicly available models have also been made. Recently, Xiao Ma generated a disproof using GPT-5.5 Pro (10). However, the transcript (11) documents an interactive process in which the human user evaluates the model’s intermediate outputs and selects proof strategies for the model to pursue. Although Ma argued that this human guidance could in principle be automated given sufficient test-time compute, the transcript in its present form represents a human-in-the-loop approach. Consequently, prior to our work, no autonomous disproof using a public model had been demonstrated.

## Our Contribution.

To avoid data contamination, we base our agent on GPT-5.5 Pro. This model was released nearly a month before OpenAI’s announcement (1), so no public proof refuting the conjecture could have been included in its training data. At inference time, web search was disabled in all trials.

Our autonomous agent uses a simple three-stage prompting pipeline: brainstorm, execute, and review. We ran eight independent trials. In each trial, the agent produced a correct or essentially correct disproof of the Erdős unit distance conjecture. The resulting proofs are diverse and do not merely reproduce earlier AI-generated proofs. The system operates efficiently, using an average of 106.7 k reasoning tokens per trial. Thus, in hindsight, such a breakthrough falls well within the capabilities of models publicly released before OpenAI’s announcement, without requiring complex scaffolding or access to models that were nonpublic at the time. We release the source code for our agent, all intermediate model outputs, and all eight generated proofs in the project repository (12).

## Materials and Methods

While termed an agent, our system has a minimalist architecture: it runs a three-round conversation, prompting the underlying model to sequentially brainstorm strategies, execute a strategy, and critically review the resulting proof.

The system prompt provides the context and objective:

**System Prompt**
The provided theorem was recently proved, and the result is now accepted by the mathematical community. Your task is to independently construct a proof.

The first prompt states the theorem to be proved and asks the model to brainstorm:

**First Prompt**
**Theorem.** There exist an absolute constant δ>0 and an infinite sequence $\left\{P_{i}\right\}$ of finite point sets in $R^{2}$ such that $|P_{i}|\to \infty$ and $u\left(P_{i}\right)\geq {\left|P_{i}\right|}^{1+\delta }$ for all *i*, where $u\left(P_{i}\right)=\frac{1}{2}|\{\left(p,q\right)\in P_{i}\times P_{i}{:\|p-q\|}_{2}=1\}|$ is the number of unit-distance pairs.
  **Remark.** This result disproves the Erdős Unit Distance Conjecture.
  ***
  As a first step, brainstorm and propose three most promising approaches to prove this theorem. Explore each approach to sufficient depth, and then rank them from most to least promising.

The second prompt asks the model to execute the selected strategy:

**Second Prompt**
Thoroughly develop your top-ranked approach to construct a complete and rigorous proof, adapting your strategy as needed.

The third prompt asks the model to conduct a critical review and present the final proof:

**Third Prompt**
Critically examine the proof you constructed to identify and resolve any errors or logical gaps. Then, present a complete, rigorous, step-by-step proof. Clearly justify each step with sufficient detail so that an expert can verify your reasoning without needing to fill in any gaps.

Two remarks are in order. First, the prompts directly ask the model to disprove the Erdős unit distance conjecture. If it were unknown whether the conjecture is true or false, this hint could be avoided simply by running the agent twice, once to attempt a proof and once to attempt a disproof, thereby roughly doubling the test-time compute. Second, problem-specific text appears only before the *** separator in the first prompt. The reasoning instructions—brainstorming, execution, and review—are problem-agnostic. Consequently, this pipeline can be applied to other theorem-proving tasks by changing only the problem-specific text in the first prompt; problems seeking a rigorously justified final answer require minor additional modifications to the prompts.

All trials were run via the GPT-5.5 Pro API using the gpt-5.5-pro-2026-04-23 snapshot. Tools and web search were disabled. The reasoning effort parameter was set to “xhigh.” The verbosity parameter was set to “high” for the first two rounds, and left at the default (medium) setting for the third round. All other parameters, including temperature and maximum output tokens, remained at their defaults.

## Performance and Efficiency

Our agent produced correct or essentially correct proofs in all eight independent trials. This corresponds to an observed pass@1 accuracy of 100%.

Table 1 reports the mean and SD of reasoning token usage across the eight trials, with a breakdown by pipeline stage. The agent used an average of 106.7 k reasoning tokens per trial.

**Table 1.** Mean and SD of reasoning token counts across the eight trials, with a breakdown by pipeline stage

Stage	Mean	SD
Round 1: Brainstorming	42.4	16.0
Round 2: Execution	41.9	11.1
Round 3: Review	22.3	3.8
Total	106.7	22.7

Token counts are reported in thousands and rounded to the nearest hundred.

Directly comparing the efficiency of our agent with that of OpenAI’s internal model is impossible because the test-time compute used by that model remains undisclosed. However, we can establish a lower bound on the length of the internal model’s reasoning trace using OpenAI’s published abridged chain-of-thought document (13). By extracting text from this PDF and processing it through OpenAI’s official token-counting API (client.responses.input_tokens.count), we estimate that the document is approximately 113 k tokens long.

Two caveats are in order. First, text extraction from a mathematically dense PDF is imperfect, introducing a minor error in the resulting token count (we expect the relative error to be ≲5%). Up to this uncertainty, this 113 k token count serves as a lower bound because the PDF is explicitly described by OpenAI as an abridged version of the model’s chain of thought. Second, this single published sample was likely curated and may not be representative of the average reasoning token usage of OpenAI’s internal model across runs.

Nevertheless, this information provides useful context. OpenAI’s internal model reached a maximum pass@1 accuracy of 48% (Fig. 1) using an undisclosed, presumably large amount of test-time compute. By contrast, our GPT-5.5 Pro agent produced correct or essentially correct proofs in all eight trials, using an average of 106.7 k reasoning tokens per trial. Because this average is below the 113 k-token lower bound derived from OpenAI’s abridged chain of thought, our agent appears to use fewer reasoning tokens while achieving an observed pass@1 accuracy of 100%.

## Comparison of Proofs

We compare the OpenAI proof (6) [and its human-digested version (7)], the Claude Mythos proof (9), and the eight proofs generated by our agent. We refer to these eight proofs as Proof 1 through Proof 8, corresponding to their filenames in the project repository.

The task is to construct arbitrarily large finite point sets $P\subset R^{2}$ such that p,p+v∈P for many pairs (p,v) with ${\left\|v\right\|}_{2}=1$. All proofs considered here are based on sequences of number fields whose degrees tend to infinity. The Minkowski embedding maps the algebraic integers of a number field onto a lattice in a high-dimensional Euclidean space. All but Proof 7 select a finite set of algebraic integers corresponding to the lattice points in a bounded region. Proof 7 instead selects a finite set of algebraic integers, each of which is a sum of algebraic units. Finally, a chosen field embedding maps the selected algebraic integers to real or complex numbers, which, after any necessary rescaling, provide coordinates for points in *P*.

The proofs fall into two groups according to the types of number fields used to construct many unit vectors *v*. The OpenAI proof and Proofs 1 to 6, 8 form the first group; they use CM (complex multiplication) fields in which a fixed rational prime splits completely. The Claude Mythos proof and Proof 7 form the second group; they use mixed-signature fields—fields with both real and complex embeddings.

Within each group, the proofs also differ in how they select the finite set of algebraic integers from which *P* is constructed. Among the proofs that make this selection by intersecting the corresponding lattice with a bounded region, the OpenAI proof and Proofs 2, 3, 5 use polydiscs (products of disks); Proof 4 uses a Euclidean ball, a choice also proposed by Eric Naslund (14); Proofs 1, 6, 8 use products of intervals; the Claude Mythos proof uses a product of intervals and disks. The OpenAI proof and Proofs 2, 3, 5 share one overarching structure, while Proofs 1, 6, 8 share another. Within each collection, however, the proofs differ in their specific mathematical realizations.

The Claude Mythos proof and Proofs 1, 6, 8 construct *P* as a Cartesian product X×X, where X⊂R is finite.

### AI Use Statement.

GPT-5.5, GPT-5.5 Pro, and Gemini 3.1 Pro Preview assisted me in understanding dense mathematical literature and the proofs generated by the agent. GPT-5.5 Codex generated the code for the agent and estimated the token count of OpenAI’s abridged chain-of-thought document (13). Gemini 3.1 Pro Preview drafted this paper based on materials I provided and assisted with editing. I independently verified all proofs and take full responsibility for the paper.

## Data Availability

The agent’s source code, all intermediate model outputs, and all eight generated proofs are available on GitHub (https://github.com/yichenhuang/unit-distance) (12).

## Footnotes

^*^As noted in ref. 2, Lijie Chen used the model; Mark Sellke and Mehtaab Sawhney verified the proof.

^†^After this paper was submitted to arXiv (which did not accept the submission) and to HAL (hal-05643551) and after our code and outputs were released on GitHub, Anthropic publicly released Claude Fable 5—officially described as a “Mythos-class” model—on June 9, 2026. However, the exact Claude Mythos model and the agent used by Alpöge remain nonpublic.
